# Cyclosporine A Inhibits Viral Infection and Release as Well as Cytokine Production in Lung Cells by Three SARS-CoV-2 Variants

**DOI:** 10.1128/spectrum.01504-21

**Published:** 2022-01-05

**Authors:** Claudio Fenizia, Silvia Galbiati, Claudia Vanetti, Riccardo Vago, Mario Clerici, Carlo Tacchetti, Tiziana Daniele

**Affiliations:** a Department of Pathophysiology and Transplantation, Milano University Medical School, Milan, Italy; b Department of Biomedical and Clinical Sciences “L Sacco,” Milano University Medical School, Milan, Italy; c Complication of Diabetes Unit, Diabetes Research Institute, IRCCS San Raffaele Scientific Institute, Milan, Italy; d Urological Research Institute, IRCCS San Raffaele Scientific Institute, Milan, Italy; e Vita-Salute San Raffaele University, Milan, Italy; f IRCCS Don Carlo Gnocchi Foundation, Milan, Italy; g Cancer Imaging Unit, Experimental Imaging Centre, IRCCS San Raffaele Scientific Institute, Milan, Italy; Shandong First Medical University

**Keywords:** SARS-CoV-2, COVID-19, infection, repositioning, CsA, cytokine, interleukin, B.1.1.7, P.1, variants, cyclophilin A, cyclosporine A

## Abstract

In December 2019, a new severe acute respiratory syndrome coronavirus 2 (SARS-CoV-2) started spreading worldwide causing the coronavirus disease 2019 (COVID-19) pandemic. The hyperactivation of the immune system has been proposed to account for disease severity and death in COVID-19 patients. Despite several approaches having been tested, no therapeutic protocol has been approved. Given that Cyclosporine A (CsA) is well-known to exert a strong antiviral activity on several viral strains and an anti-inflammatory role in different organs with relevant benefits in diverse pathological contexts, we tested its effects on SARS-CoV-2 infection of lung cells. We found that treatment with CsA either before or after infection of CaLu3 cells by three SARS-CoV-2 variants: (i) reduces the expression of both viral RNA and proteins in infected cells; (ii) decreases the number of progeny virions released by infected cells; (iii) dampens the virus-triggered synthesis of cytokines (including IL-6, IL-8, IL1α and TNF-α) that are involved in cytokine storm in patients. Altogether, these data provide a rationale for CsA repositioning for the treatment of severe COVID-19 patients.

**IMPORTANCE** SARS-CoV-2 is the most recently identified member of the betacoronavirus genus responsible for the COVID-19 pandemic. Repurposing of available drugs has been a “quick and dirty” approach to try to reduce mortality and severe symptoms in affected patients initially, and can still represent an undeniable and valuable approach to face COVID-19 as the continuous appearance and rapid diffusion of more “aggressive”/transmissible variants, capable of eluding antibody neutralization, challenges the effectiveness of some anti-SARS-CoV-2 vaccines. Here, we tested a known antiviral and anti-inflammatory drug, Cyclosporine A (CsA), and found that it dampens viral infection and cytokine release from lung cells upon exposure to three different SARS-CoV-2 variants. Knock down of the main intracellular target of CsA, Cyclophilin A, does not phenocopy the drug inhibition of viral infection. Altogether, these findings shed new light on the cellular mechanisms of SARS-CoV-2 infection and provide the rationale for CsA repositioning to treat severe COVID-19 patients.

## INTRODUCTION

In December 2019, a new severe acute respiratory syndrome coronavirus 2 (SARS-CoV-2) started spreading worldwide causing the coronavirus disease 2019 (COVID-19) pandemic. Patients affected by this pathology present various clinical manifestations, and complications might affect different organs, including lung, liver, kidney, heart, and brain (1–3). An excessive activation of the immune response has been proposed to account for disease severity (affecting around 5% of patients) and death in COVID-19 patients. Host cell infection by SARS-CoV-2 triggers the induction of inflammatory cytokines, including IL1β, IL-2, IL-6, IL-7, IL-8, IL-10, interferon (IFN)-γ, IFN-γ inducible protein (IP)-10/CXCL10, granulocyte colony-stimulating factor (G-CSF), monocyte chemoattractant protein (MCP)-1/C-C motif chemokine ligand 2 (CCL2), macrophage inflammatory protein (MIP) 1α, and tumor necrosis factor (TNF) α, which in turn recruit macrophages and neutrophils to the site of infection culminating in a “cytokine storm” or “cytokine release syndrome” (4–6). Such unbalanced exacerbated immune response is accompanied by a reduction of type I interferons (IFN-Is), either as result of SARS-CoV-2 immune escape mechanism or due to the production of auto-immune antibodies (7–10). Although a wide consensus has not been reached yet, multiple publications report that the intensity of such cytokine release syndrome correlates with disease severity (11–13).

Cyclosporine A (CsA), a natural cyclic peptide of 11 amino acids, is an inhibitor of cyclophilins (proteins belonging to the superfamily of immunophilins) known to prevent T cell activation via the formation of a tri-partite complex that includes Cyclophilin A (CyPA), CsA and calcineurin. The subsequent inhibition of NFAT translocation to the nucleus (14, 15) and the inhibition of CyPA binding to interleukin-2 tyrosine kinase (Itk) that remains constitutively activated (16), reduces the immune response mediated by T cells (17). Moreover, CsA has been shown to affect also innate immunity (recently reviewed in [18]). Therefore, CsA exerts both immunosuppressive and anti-inflammatory activities.

CsA has also been reported to interfere with viral infection and replication of different strains, including hepatitis B virus (HBV), hepatitis C virus (HCV), hepatitis D virus (HDV), influenza virus, cytomegalovirus, rotavirus, human immunodeficiency virus (HIV) and coronaviruses (19, 20), including SARS-CoV and Middle East respiratory syndrome (MERS)-CoV (21, 22).

We investigated the effects of CsA on SARS-CoV-2 infection in CaLu3 cells, a human pulmonary cell line; results showed that CsA hampers both viral infectivity and the production of pro-inflammatory cytokines by three different variants of SARS-CoV-2, suggesting a potential exploitation of this drug in the therapy of COVID-19.

## RESULTS

SARS-CoV-2 is able to enter different organs, thus we tested four different human cell lines (A549 and CaLu3 from lungs, HepG2 from liver, and CaCo2 from intestine) as model systems to study viral infection, whereas we used Vero E6 cells (from African green monkey kidney, a standard system for laboratory propagation of viruses) to initially expand SARS-CoV-2. In accordance with previous findings (23), CaLu3 pulmonary cells were found to be the most efficiently infected and, therefore, used as the model system for all the experiments reported here.

### CsA impairs SARS-CoV-2 RNA replication and protein synthesis, and the production of progeny viral particles.

**FIG 1:**
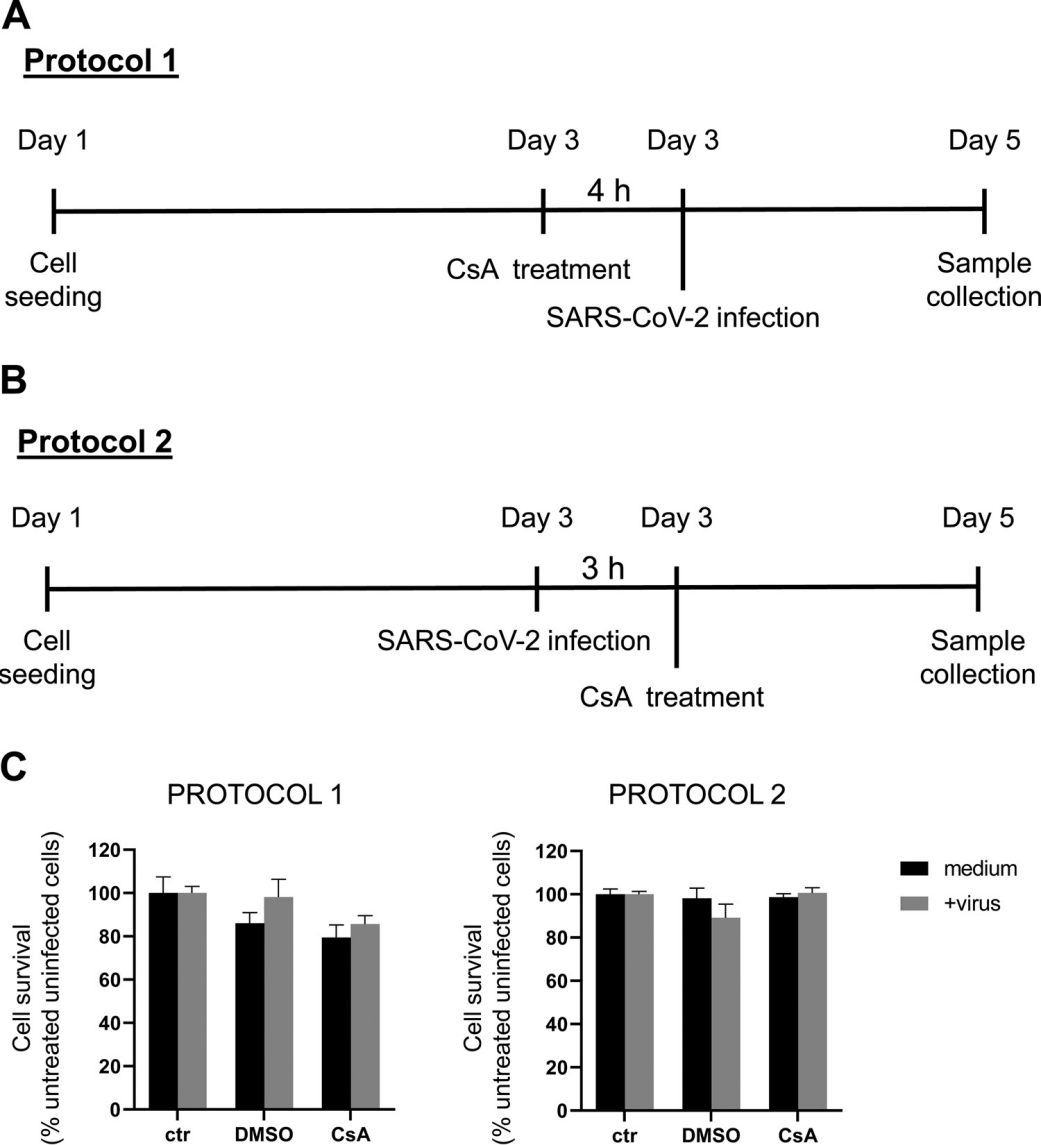
CsA treatment and/or SARS-CoV-2 infection does not affect CaLu3 cells viability. (A, B) Schematic representation of the experimental protocols, as indicated. CaLu3 cells were treated with 10 μM CsA either 4 h before (protocol 1, A) or 3 h after (protocol 2, B) SARS-CoV-2 infection. Cells were analyzed 48 hpi. (C) Cell survival was assessed by MTT assay. Data are normalized to the control uninfected sample. Graphs show mean ± SD out of five technical replicates. One experiment is shown as representative of two.

**FIG 2:**
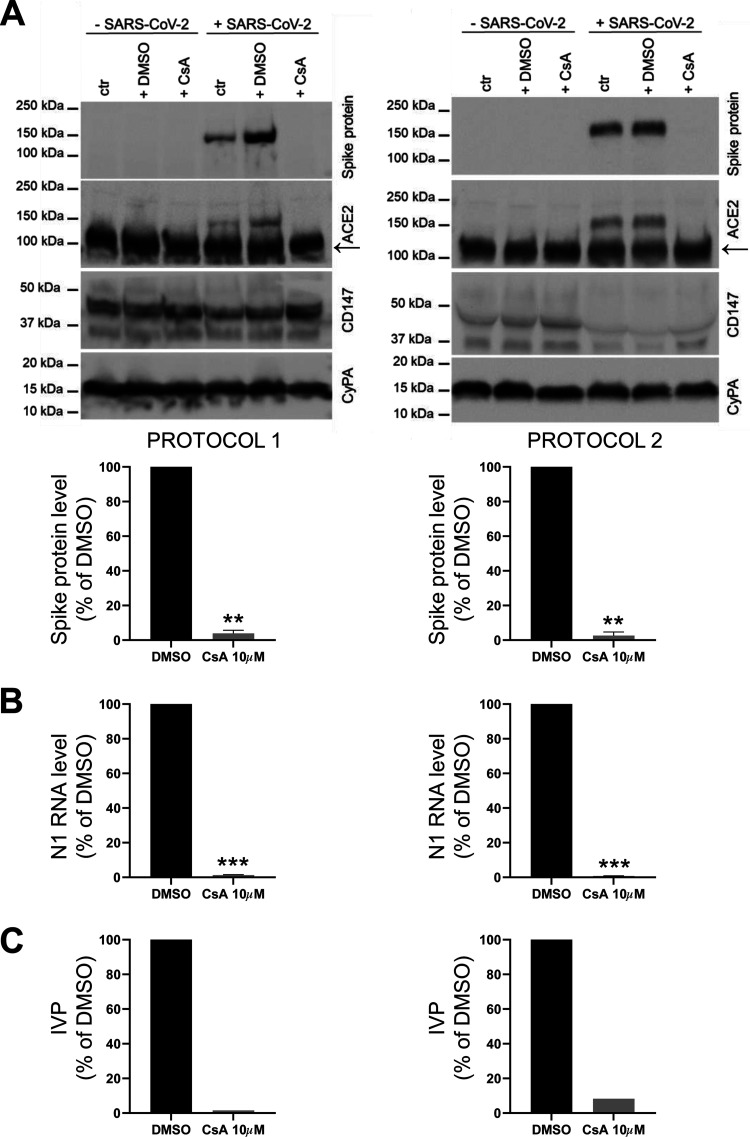
CsA impairs SARS-CoV-2 ability to infect CaLu3 cells when administered either before or after viral infection. CaLu3 cells were treated with 10 μM CsA either before (protocol 1, left) or after (protocol 2, right) SARS-CoV-2 infection. Samples were collected 48 hpi. (A) Spike protein level was evaluated by WB analysis. ACE2 labeling was detected after Spike protein immunostaining on the same membrane. Arrow indicates ACE2 specific band. CyPA was used as the loading control. Spike protein levels in CsA-treated samples were normalized to the levels in DMSO-treated controls. Graphs show mean ± SEM out of three independent experiments. **, *P < *0.01 Student's *t* test. (B) N1 RNA levels were determined by ddPCR analysis and normalized to total RNA. N1 levels in CsA-treated samples were normalized to the levels in DMSO-treated controls. Graphs show mean ± SEM out of three independent experiments. ***, *P < *0.001 Student's *t* test. (C) Viral titer of cell supernatants from one experiment was quantified by means of TCID_50_ determination. Infecting viral particles (IVP) in CsA-treated cell supernatants were normalized to those in DMSO-treated cell supernatants.

CaLu3 cells were treated with 10 μM CsA both before (protocol 1, Fig. 1A) or after (protocol 2, Fig. 1B) infection with 0.05 MOI SARS-CoV-2; samples were collected 48-h postinfection (hpi). CaLu3 cell survival upon CsA treatment either in the presence or in the absence of SARS-CoV-2 was assessed by 3-(4,5-dimethylthiazol-2-yl)-2,5-diphenyltetrazolium bromide (MTT) colorimetric assay. While neither viral infection nor drug treatment affected cell viability (Fig. 1C), western blotting (WB) analysis showed that CsA-treated cells expressed significantly lower levels of SARS-CoV-2 Spike protein compared to control dimethyl sulfoxide (DMSO)-treated cells independently on whether the drug was administered before (protocol 1, 3.90 ± 1.84% of DMSO-treated samples) or after (protocol 2, 2.66 ± 2.05% of DMSO-treated samples) viral infection (Fig. 2A).

Accordingly, immunofluorescence analysis confirmed that the number of infected CaLu3 cells was reduced by drug treatment in both experimental settings (Fig. S1), as revealed by anti-Spike protein labeling. Moreover, also the intracellular viral load, measured by ddPCR analysis of the RNA levels of nucleocapside (N1), was significantly decreased in cells treated with CsA either before (protocol 1, 1.32 ± 0.19% of DMSO-treated samples) or after (protocol 2, 0.80 ± 0.10% of DMSO-treated samples) infection with SARS-CoV-2 (Fig. 2B). To test whether CsA treatment also affects the production of an infectious progeny of SARS-CoV-2, we analyzed the levels of N1 RNA in the supernatant and quantified the virus titer by means of TCID_50_ determination (Fig. 2C). We found that CsA treatment dampens the number of released infectious viral particles in both experimental conditions.

Altogether, these findings show that CsA interferes with SARS-CoV-2 viral RNA replication, protein synthesis as well as with the assembly and release of new virions.

**FIG 3:**
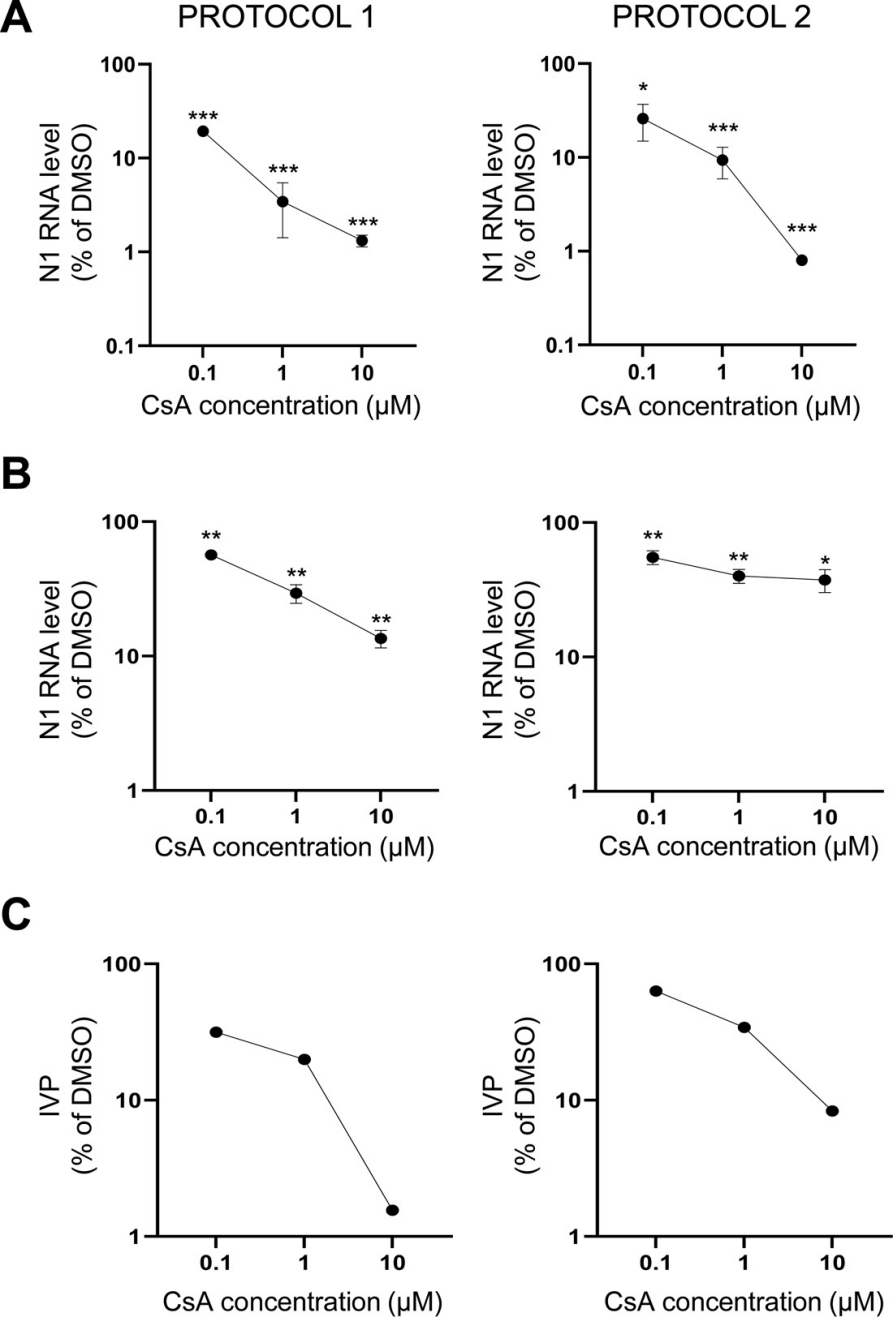
CsA is effective also at concentrations comparable with those reached in the blood of transplanted patients. CaLu3 cells were treated with 10, 1, or 0.1 μM CsA either before (protocol 1, left) or after (protocol 2, right) SARS-CoV-2 infection. Samples were collected 48 hpi. Dose-response graphs show mean ± SEM out of three independent experiments. (A, B) N1 levels in cells extracts (A) and supernatants (B) were analyzed by ddPCR analysis and normalized to total RNA. N1 levels in CsA-treated samples were normalized to the levels in DMSO-treated controls. ***, *P < *0.001; **, *P < *0.01; *, *P < *0.5, Student's *t* test. (C) Viral titer of cell supernatants from one experiment was quantified by means of TCID_50_ determination. Infecting viral particles (IVP) in CsA-treated cell supernatants were normalized to those in DMSO-treated cell supernatants.

To test the efficacy of this drug at concentrations similar to those reached in the blood of transplanted patients (approximately 800 ng/mL [about 0.67 μM] in the first 2 to 4 h after administration—peak value-, and around 100 ng/ml [about 0.083 μM] 10-h postadministration—trough level_[24]), we treated CaLu3 cells with 1 and 0.1 μM CsA (Fig. 3). ddPCR and TCID_50_ analysis revealed that in both experimental settings all three concentrations of CsA reduce viral infection and release (with increasing efficacy augmenting drug dose), when compared with control DMSO-treated samples. In particular, CsA administration before infection appears more effective than after (protocol 1 vs protocol 2). Results also showed a good correlation between the levels of virus RNA in the cell extracts (Fig. 3A) and the number of released infectious viral particles (IVP) in the supernatants (Fig. 3C) of CsA-treated cells. On the other hand, we found higher levels of viral RNA (Fig. 3B) compared with IVP (Fig. 3C) in the supernatants in all the CsA-treated conditions, possibly due to non-infectious viral particle release and/or cell death.

Altogether, these results suggest that CsA is effective even at lower concentrations that are compatible with the clinical practice.

### CsA exerts antiviral activity also on B.1.1.7 and P.1 SARS-CoV-2 variants.

**FIG 4:**
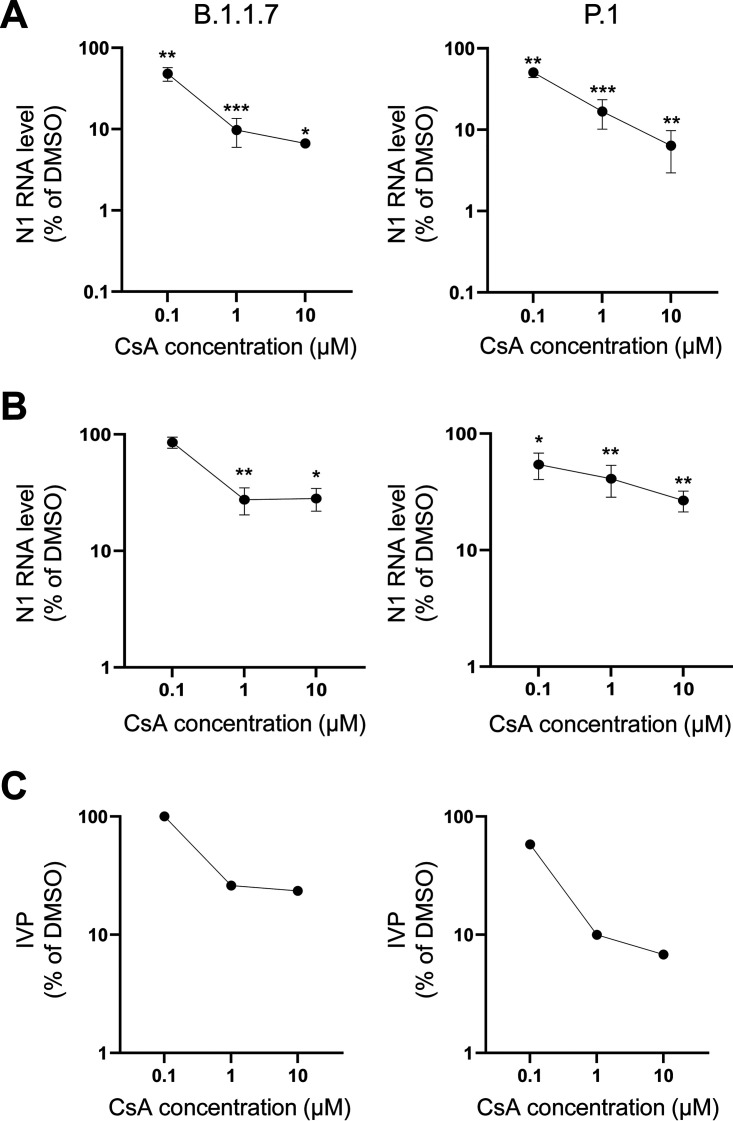
CsA exerts antiviral activity also on B.1.1.7 and P.1 SARS-CoV-2 variants. CaLu3 cells were treated with 10, 1 or 0.1 μM CsA after infection with B.1.1.7 (left) and P.1 (right) SARS-CoV-2 strains. Samples were collected 48 hpi. Dose-response graphs show mean ±SEM out of three independent experiments. (A-B) N1 levels in cells extracts (A) and supernatants (B) were analyzed by ddPCR analysis and normalized to total RNA. N1 levels in CsA-treated samples were normalized to the levels in DMSO-treated controls. ***, *P < *0.001; **, *P < *0.01; *, *P < *0.5, Student's *t* test. (C) Viral titer of cell supernatants from one experiment was quantified by means of TCID_50_ determination. Infecting viral particles (IVP) in CsA-treated cell supernatants were normalized to those in DMSO-treated cell supernatants.

The appearance and rapid diffusion of more “aggressive”/transmissible variants of SARS-CoV-2, due to an increased affinity for ACE2 receptor and resistance to antibody neutralization (25, 26), prompted us to test the efficacy of CsA also on these new strains. In particular, we analyzed the viral load in samples treated with the three concentrations of CsA after infection (protocol 2) with either B.1.1.7 (U.K variant, alpha) or P.1 (Brazil lineage, gamma) SARS-CoV-2 by ddPCR and found that CsA treatment significantly reduced virus RNA replication in the cells at all the concentrations tested for both SARS-CoV-2 strains (Fig. 4A). Results showed a good correlation between the levels of viral RNA and IVP in the supernatants of CsA-treated B.1.1.7-infected cells, suggesting that this variant releases mainly infecting virions. By contrast, we found higher levels of viral RNA (Fig. 4B) compared with IVP (Fig. 4C) in the supernatants of CsA-treated P.1-infected cells, possibly due to non-infectious viral particle release and/or cell death.

Altogether, these results show that CsA exerts antiviral activity also on SARS-CoV-2 P.1 and B.1.1.7 variants. Moreover, our findings suggest that the lowest (0.1 μM) CsA concentration is more effective on the EU strain than on the SARS-CoV-2 variants (Fig. 3 and 4).

### CsA diminishes the synthesis of virus-induced cytokines in lung cells.

**FIG 5:**
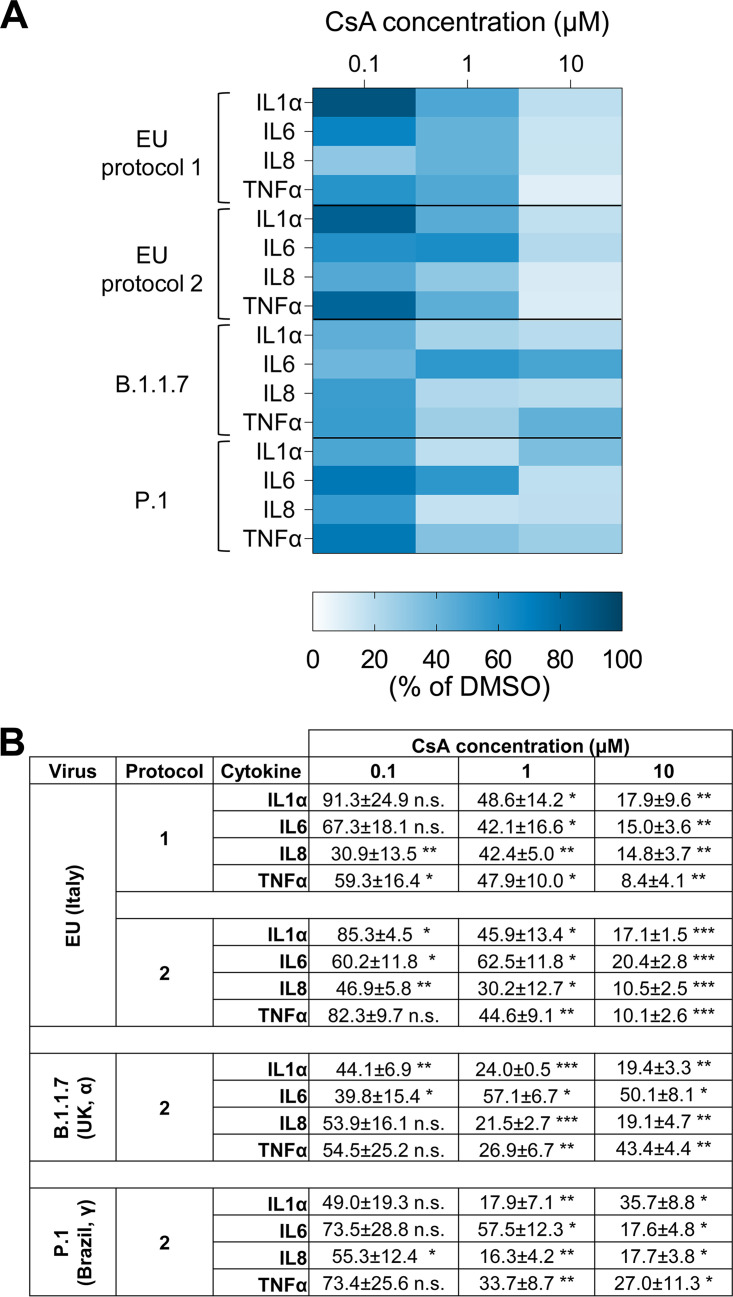
CsA dampens cytokines synthesis induced by SARS-CoV-2 infection. CaLu3 cells were treated with 0.1, 1, or 10 μM CsA or vehicle (DMSO, as control) either before (protocol 1) or after (protocol 2) infection with three SARS-CoV-2 strains (as indicated). Samples were collected 48 hpi. Cytokines levels were assessed by rtPCR analysis and normalized to GAPDH. Cytokine RNA levels in CsA-treated samples were normalized to the levels in DMSO-treated controls. (A) RNA levels of cytokines (mean values) are shown as a color scale from light blue to blue (Heatmap). (B) Table shows mean ± SEM out of three independent experiments ***, *P < *0.001; **, *P < *0.01; *, *P < *0.5; n.s, *P > *0.5 Student's *t* test.

SARS-CoV infection was reported to induce the synthesis of epithelial cytokines in CaLu3 cells (27); therefore we decided to investigate whether also SARS-CoV-2 triggered cytokine production and to test the effect of CsA treatment. To this purpose we analyzed the levels of a panel of relevant cytokines, including IL1α (which triggers the recruitment of hematopoietic cells that in turn amplify and induce IL1α production in a positive feedback loop sustaining inflammation [28]), TNF-α (a strong pro-inflammatory cytokine [29]), IL-8 (which plays an important role in both neutrophil recruitment and activation [30]), and IL-6 (which exerts pro-inflammatory activities in a context-dependent manner [31]), which are involved in the cytokine release disease reported in COVID-19 patients, in the cellular extracts of CaLu3 treated with CsA either before (protocol 1) or after (protocol 2) SARS-CoV-2 infection by rtPCR analysis. Results showed that CsA significantly reduced the amount of cytokine RNA synthesized upon viral infection in both experimental settings and by all SARS-CoV-2 strains at the higher concentrations tested (10 and 1 μM, Fig. 5). Moreover, we found that CsA appears to exert an “all or none” effect on cytokine RNA production induced by B.1.1.7 and P.1 variants (effective at similar levels at 10 and 1 μM and almost ineffective at 0.1 μM), whereas the drug displays a more dose-dependent effect on cytokine induction promoted by the EU strain of SARS-CoV-2.

Altogether, these findings support an anti-inflammatory activity of CsA on lung cells infected by SARS-CoV-2.

### Cyclophilin A silencing does not phenocopy CsA effects on SARS-CoV-2 infection.

**FIG 6:**
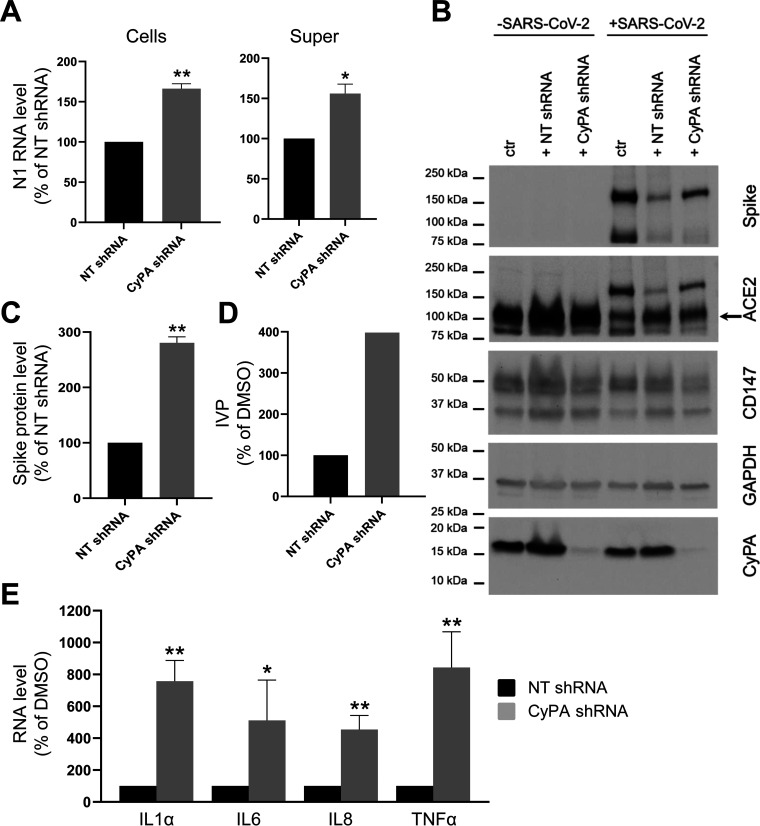
CyPA knock down favors SARS-CoV-2 infection. CaLu3 cells were either not transduced (ctr) or transduced with a nontargeting (NT) or a CyPA-specific shRNA for 12 days before infection with SARS-CoV-2. Samples were collected 48 hpi. (A) N1 levels in cells and supernatants (super) were analyzed by ddPCR analysis and normalized to total RNA. N1 levels in CyPA-silenced cells were normalized to the levels in NT shRNA-transduced control. Graphs show mean ± SEM out of three independent experiments. **, *P < *0.01; *, *P < *0.05 Student's *t* test. (B) One experiment is shown as representative of three. CD147 labeling was performed after GAPDH immunostaining on the same membrane. ACE2 labeling was detected after Spike protein immunostaining on the same membrane. Arrow indicates ACE2 specific band. (C) Spike protein expression was evaluated by WB analysis. GAPDH was used as the loading control. Spike protein levels in CyPA-silenced cells were normalized to the levels in NT shRNA-transduced control. Graphs show mean ± SEM out of three independent experiments. **, *P < *0.01, Student's *t* test. (D) Viral titer of cell supernatants from one experiment was quantified by means of TCID_50_ determination. Infecting viral particles (IVP) in CyPA-silenced cell supernatants were normalized to those in NT shRNA-treated cell supernatants. (E) Cytokines RNA levels were assessed by rtPCR analysis and normalized to GAPDH. Data from CyPA-silenced cells were normalized to those in NT shRNA-treated control. Graph shows mean ± SEM out of three independent experiments. **, *P < *0.01; *, *P < *0.1. Student's *t* test.

CyPA is the main intracellular target of CsA (32) and it acts as an intracellular: (i) chaperone during viral replication for different viruses (33, 34); and (ii) sensor that favors viral infection (35, 36). Furthermore, CyPA has been identified as the top ranked hit in a meta-analysis study of host genes implicated in COVID-19 (37), and CsA ability to impair SARS-CoV-2 infection in CaLu3 cells has been suggested to depend on its action on cyclophilins (23). Thus, we investigated the role of CyPA in SARS-CoV-2 entry into host cells, exploiting a genetic approach. We silenced CyPA expression in CaLu3 cells before viral infection by transduction with a specific short hairpin RNA (shRNA): CyPA knock down efficiency was 96.0% ± 0.7% of control NT shRNA-transduced cells at the RNA and 95.1% ± 1.0% of control NT shRNA-transduced cells at the protein level. We evaluated SARS-CoV-2 RNA load in both cells and supernatants by ddPCR analysis, and found that N1 levels were increased in CyPA-knocked down cells (166.3% ± 6.1% and 156.1% ± 11.6% of control NT shRNA-transduced cells, respectively, Fig. 6A). Furthermore, we assessed the levels of viral Spike protein by WB analysis (Fig. 6B and C), and found that silencing of CyPA augmented its levels (280.6% ± 10.7% of control NT shRNA-transduced cells, Fig. 6C). To test whether knock down of CyPA also affects the production of an infectious progeny of SARS-CoV-2, we quantified the virus titer by means of TCID_50_ determination and found that CyPA silencing increases the number of released IVP (Fig. 6D).

Because reduction of viral infection impairs cytokine production (Fig. 5), we analyzed the RNA levels of IL1α, IL-6, IL-8, and TNF-α in Calu3 cells treated with CyPA-specific shRNA before viral infection, and found that CyPA knockdown augmented their expression (IL1α, 757.1 ± 129.9; TNF-α, 843.2 ± 224.1; IL-6, 511.8 ± 253.1; IL-8, 454.7 ± 88.5% of control NT shRNA-treated cells [Fig. 6E]).

Altogether, these results suggest that CyPA works as a negative modulator of SARS-CoV-2 infection, and therefore, that CsA inhibitory effects on both viral replication and cytokine production have to be ascribed to a different molecular mechanism.

## DISCUSSION

In December 2019, the newly identified coronavirus SARS-CoV-2 started spreading worldwide causing the COVID-19 pandemic. Several approaches have been suggested and tested for the treatment of COVID-19 patients, but till now no therapeutic protocol has been approved. The lack of solid therapeutic approaches for the treatment of COVID-19 patients led to the idea of verifying whether CsA, a molecule endowed with potent antiviral and anti-inflammatory activities, could play a role in this scenario (38–49).

Some preliminary data obtained in Vero E6 cells suggested that CsA might interfere with SARS-CoV-2 infection (50); thus we tested the drug effects in the physiological context of pulmonary CaLu3 cells. We found that *in vitro* CsA exerts two different effects: (i) it impairs viral infection, replication and release, and (ii) it diminishes the virus-induced synthesis of cytokines by CaLu3 cells (as previously reported for SARS-CoV [27]). Recently, the ability of CsA to interfere with SARS-CoV-2 infection has been reported also in CaLu3 cells, and suggested to rely on its action on cyclophilins (23). Here, we provide additional evidence that CsA interferes with viral infection and dampens subsequent epithelial cytokines production also by B.1.1.7 (alpha) and P.1 (gamma) variants of SARS-CoV-2, thus suggesting that the “emerged” viral mutations have not affected the drug-targeted molecular machinery. Moreover, by exploiting a genetic approach, we show that CsA effect is not mediated by its main intracellular target, CyPA.

CsA is known to exert several antiviral activities, including the inhibition of genome replication and particles assembly, as well as the regulation of the activity of host restriction factors (20, 21), mainly by its inhibitory functions on cyclophilins, in particular CyPA. Indeed, it plays an essential role in promoting viral infection exerting its functions both inside and outside host cells. In particular, CyPA (i) acts as an intracellular chaperone during viral replication for different viruses (33, 34); (ii) behaves as an intracellular sensor that favors viral infection (hampering the innate immune response [35], and regulating the sensitivity to host restriction factors [36]); and (iii) partakes in target cells invasion by HIV-1 and SARS-CoV (51–53), but not by SARS-CoV-2 (54), mediating virus binding to the CD147 receptor. Furthermore, CyPA has been recently reported as the top ranked hit in a meta-analysis study of host genes implicated in COVID-19 (37). Interestingly, our experiments with CyPA-silenced cells provide evidence that CyPA works as a negative modulator of SARS-CoV-2 infection, as already described for influenza virus and rotavirus (33, 34). Therefore, our results suggest that CsA ability to impair viral activity has to be ascribed to other cyclophilins or to a different molecular mechanism.

As for the former hypothesis, two additional candidates should be considered among cyclophilins, namely, CyPB and CyPD. CyPB (found in the endoplasmic reticulum) is the only other cyclophilin expressed at detectable levels inside the cells in addition to CyPA, which instead accounts for 0.1% to 0.5% of the total cellular protein content. CyPB has been reported to play a role in host infection by HCV (55) and HIV-1 (56), in the latter case modulating viral translocation into the nucleus in a CsA-independent manner (57). CyPD, an immunophilin localized on the inner membrane of mitochondria, has been shown to partake in coronavirus (HCoV-OC43) infection in a CsA-dependent manner (58, 59).

As for the latter hypothesis, CsA has been proposed recently to be able to interfere with SARS-CoV-2 entry. Indeed, Prasad et al. unraveled that CsA can bind and inhibit two classes of host proteases, namely, TMPRSS2 and Cathepsins, using a computational approach, and suggested that the drug might work also on the initial phases of SARS-CoV-2 infection of target cells (60). Moreover, some data provided by Dittmar et al. could support an involvement of TMPRSS2 in CsA mechanism of action as they show that cell treatment with camostat appears to phenocopy the effects of CsA on SARS-CoV-2 infection (23). Our findings that CsA functions in both experimental settings (protocol 1, CsA treatment before infection, and protocol 2, CsA treatment after infection) are compatible with either hypothesis. Indeed, CsA is a membrane permeable drug, thus it might work on already infected cells inhibiting new virions assembly and/or blocking new cell infection by viral progeny.

CsA exerts anti-inflammatory activities as well, both inside and outside the cell. Intracellularly, CsA sequesters cyclophilins from binding to calcineurin, thus avoiding NFAT translocation to the nucleus and downstream cytokine synthesis (18). Extracellularly, CsA binds secreted CyPA or CyPB and impairs their chemotactic activity on eosinophils, neutrophils, T lymphocytes which is driven by recognition of the CD147 receptor (61, 62), and dampens the inflammatory response in an animal model of human acute lung injury (63). Altogether, these observations suggest that extracellular CyPA plays an essential role in inflammation in different contexts, and that its targeting might represent an effective way to reduce leukocyte redistribution to inflamed tissues and local production of cytokines (64). This hypothesis is strengthened by the finding that under mechanical ventilation airway epithelial cells actively secrete CyPA that is responsible for cytokine-driven leukocyte-mediated acute lung injury in mice, and that this phenotype is reverted upon treatment with a cyclosporine derivative (65). Moreover, extracellular CyPA levels have been found upregulated in the bronchoalveolar lavage fluids of patients with ARDS (65).

Thus, the anti-inflammatory activity of CsA might be useful to dampen the cytokine storm, possibly delaying the progression toward acute respiratory distress syndrome (ARDS) and/or a systemic inflammatory condition as observed in severe COVID-19. Indeed, patients display distinct hematological manifestations; among them lymphocytopenia characterizes approximately 70% of severe-to-critical cases. Different causes have been proposed to account for this clinical manifestation: immune exhaustion/senescence, massive recruitment of immune cells to tissues, and decreased cell production due to uncoordinated cytokine signaling (66–68). The common denominator of all these phenomena relies on the hyperactivation of the immune system and an uncoordinated excessive cytokine signaling. In this context, our finding that CsA attenuates cytokine synthesis in lung cells could result in a reduced recruitment of immune cells to the site of infection. Indeed, regulators of the immune function have been employed as therapeutic approaches (i.e., corticosteroids, anti-IL-17 monoclonal antibodies, anti-IL-6 monoclonal antibodies). Although they are known to induce lymphocytopenia, corticosteroids can promptly recover lymphocytes count due to their anti-inflammatory properties (69). In addition, they reduce mortality and the need for invasive mechanical ventilation or oxygen alone (possibly because they promote erythroid precursors maturation into red blood cells [70]), but only in severe COVID-19 patients (71, 72), whereas treatment with dexamethasone does not show beneficial effects in patients not requiring respiratory support (72) or even an increased risk of mortality or mechanical ventilation need in patients with low levels of initial C-reactive protein (71).

As for cytokine-targeted therapies, results of randomized, placebo-controlled, blinded trials targeting either IL-1 or IL-6 pathway showed no survival benefit in COVID-19 patients ([73] and [74], respectively), unless performed in combination with cortisteroids (75, 76), suggesting that inhibiting a single cytokine pathway might not be sufficient. In this context, our findings showing that CsA dampens the production of several epithelial cytokines would support its repurposing. Furthermore, our observation of a reduction of viral titer as well as of cytokine production at concentrations compatible with CsA administration to patients would support its usage *in vivo* (as recently questioned by Solanich et al. [77]).

Despite we are well aware of its potent immunosuppressive activity, we reckon that repositioning of CsA should be considered for the treatment of COVID-19 patients, upon the identification of the proper/best therapeutic window (78, 79). Indeed, some clinical evidences have been collected retrospectively on the treatment of severe COVID-19 patients with CsA; results showed no additional risks in face of reduced mortality (80). Along this line, a phase I clinical trial testing the ability of CsA to prevent cytokine storm onset in patients with moderate COVID-19 has been started in 2020 and is still ongoing (NCT04412785).

Our findings showing that CsA exerts both antiviral and anti-inflammatory activities on three different variants of SARS-CoV-2 with similar efficacy would suggest that this drug exploits a conserved mechanism and therefore might be useful in the therapy of COVID-19. By contrast, the effectiveness of some anti-SARS-CoV-2 vaccines appears to be reduced by certain viral mutations and thus novel alternative therapeutic approaches might be essential in the clinical management of COVID-19 patients (81–85). Finally, the well-known inhibitory activity of CsA on different viruses together with the results reported here demonstrating its efficacy on different SARS-CoV-2 variants support the relevance of this drug beyond the current pandemic.

## MATERIALS AND METHODS

### Antibodies and reagents.

Antibody anti-CD147 was from Santa Cruz Biotechnology (Dallas, TX, USA). Antibody anti-Spike protein antibody was from Genetex (Alton Pkwy Irvine, CA, USA). Antibodies anti-ACE2, anti-GAPDH, and anti-CyPA were from Abcam (Cambridge, Cambridgeshire, UK). HRP-conjugated secondary antibodies were purchased from Cell Signaling Technology (Danvers, MA, USA) unless otherwise stated. Hoechst 33542, Alexa 488-, and Alexa 546-conjugated secondary antibodies were obtained from Molecular Probes (Life Technologies by Thermo Scientific, Waltham, MA, USA). CsA, DMSO, HEPES, MTT, Tris, Glycine, SDS, Tween 20, saponin, NH_4_Cl, and bovine serum albumin (BSA) were purchased from Sigma (by Merck, Kenilworth, NJ, USA). All cell culture reagents were from Thermo Scientific. All chemical reagents were of analytical grade or higher, and purchased from Sigma unless otherwise specified.

### Cell culture, infection, and treatments.

Vero E6 (CRL-1586, African green monkey kidney epithelial cells), A549 (CCL-185, human epithelial cells from lung carcinoma), HepG2 (HB-8065, human epithelial cells from liver carcinoma), CaCo2 (HTB-37, human epithelial cells from colorectal adeno-carcinoma), and CaLu3 (HTB-55, human epithelial cells from lung adenocarcinoma) were purchased from American Type Culture Collection (ATCC, Manassas, VA, USA). Vero E6 cells were grown in DMEM high glucose, 2 mM Glutamax, PenStrep, 10% FBS, 1 mM HEPES, and 1 mM sodium pyruvate; A549 cells were grown in DMEM high glucose, 2 mM Glutamax, PenStrep, 10% FBS, 50 μM beta-mercaptoethanol, and 1 mM sodium pyruvate; HepG2 and Hek293T cells were grown in DMEM high glucose, 2 mM Glutamax, PenStrep, and 10% FBS; CaCo2 cells were grown in DMEM high glucose, 4 mM Glutamax, PenStrep, 20% FBS, 1% NEAA, and 1 mM sodium pyruvate; Calu3 cells were grown in DMEM high glucose, 2 mM Glutamax, PenStrep, 10% FBS, and 1% NEAA. Cells were grown at 37°C in 5% CO2 and at 98% humidity. Cells were routinely checked for mycoplasma contamination by PCR test.

SARS-CoV-2 Virus Human 2019-nCoV strain 2019-nCoV/Italy-INMI1, Rome, Italy was purchased from Integrated DNA Technologies (IDT, Coralville, IA, USA). SARS-CoV-2 B.1.1.7 and P.1 lineages were a kind gift of Davide Mileto, Clinical Microbiology, Virology and Bio-emergence Diagnosis, ASST Fatebenefratelli-Sacco, Department of Biomedical and Clinical Sciences, University of Milan, Milan, Italy. The European (EU) SARS-CoV-2 Virus (Human 2019-nCoV strain 2019-nCoV/Italy-INMI1) was used in the majority of the experiments, unless otherwise specified (see Table 1 for features of the viral strains used in this study). All the experiments with SARS-CoV-2 virus were performed in BSL3 facility (Department of Biomedical and Clinical Sciences “L. Sacco,” Milano University Medical School); virus was inactivated according to institutional safety guidelines, before samples analysis outside BSL3 area.

**TABLE 1:** Features of SARS-CoV-2 strains used in this study

Lineage	D614G	N501Y	E484K	P681H	TCID_50_/mL
EU (Italy)	+^*a*^	−	−	−	3.3*10^5^
B.1.1.7 (UK, α)	+	+	−	+	6.3*10^6^
P.1 (Brazil, ϒ)	+	+	+	−	7.9*10^5^

a +, mutation present; −, mutation absent.

In order to obtain the viral stock, SARS-CoV-2 was expanded on the human cell line CaCo2 and infectious viral particles concentration was assessed by TCID_50_. Briefly, Calu3 were seeded at 2 × 10^4^ cells per well in a 96-well plate. Eleven 1:10 serial dilutions of the viral stock were performed in 2% FBS medium. For each dilution, eight wells were infected. Eight wells were left uninfected as control. Three-hours postinfection (hpi), each well was thoroughly washed three times with pre-warmed PBS and the culture media replaced with 10% FBS DMEM. Optical microscope observation (ZOE Fluorescent Cell Imager, Bio-Rad Laboratories, Hercules, CA, USA) was performed daily to investigate the cytopathic effect. At 48-hpi, supernatants were removed, cells fixed by paraformaldehyde (PFA) 4% for 1 h at room temperature, then stained by 0.2% crystal violet solution. By applying the Reed-Muench method with the correction for the proportional distance (PD [86]), we were able to assess the TCID_50_ and to calculate the MOI in our experiments.

The day before, 2.5 × 10^5^ Calu3 cells were cultured in 0.5 mL of 2% FBS medium in a 24-well plate. Then, we followed two different protocols of infection, as follows:

Protocol 1: Cells were first pretreated with 10, 1, or 0.1 μM CsA (or equivalent DMSO, as mock control) diluted in complete medium in the absence of the virus. After 4 h of pretreatment, cells were challenged with 0.05 MOI of SARS-CoV-2. At 3-hpi, cells were thoroughly washed three times with pre-warmed PBS and refilled with the complete growth medium (10% FBS), including 10 μM CsA, DMSO, or plain culture medium (Fig. 1A).

Protocol 2: Cells were first infected with 0.05 MOI of SARS-CoV-2 and then, after removing the virus at 3-hpi, complete growth medium (10% FBS) with 10, 1, or 0.1 μM CsA, DMSO or plain culture medium was replenished (Fig. 1B).

At 48-hpi, CaLu3 cells were lysed for RNA or protein extraction, whereas supernatants were harvested and appropriately stored.

### Proliferation assay.

Cells were seeded in a 96-well plate and allowed to grow for 48 h before treatment. Cell viability was determined by the MTT assay after 48 h of treatment. Cells were incubated with 2 mM 3-(4,5-dimethylthiazol-2-yl)-2,5-diphenyltetrazolium bromide (MTT, Sigma) for 4 h at 37°C; then the supernatant was removed. Afterwards, formazan was extracted from cells with 100 μL of DMSO. The amount of MTT-formazan was determined by absorbance at 595 nm.

### RNA interference.

To knockdown CyPA expression, a short hairpin sequence targeting human CyPA and a non-targeting (NT) negative control shRNA were used, as described before (87).

The CyPA-specific oligonucleotide used was:

5′-CTGACTGTGGACAACTCGAAT-3′.

The NT shRNA used was:

5′-CAACAAGATGAAGAGCACCAA-3′.

Briefly, for lentivirus production, Hek293T cells were transfected with the calcium phosphate method. To this end, a mix containing 10 μg of either lentiviral plasmid DNA vector, 6.5 μg of packaging vector Δr 8.74, 3.5 μg of Env VSV-G, 2.5 μg of REV, ddH_2_O to 450 μL, 50 μL of 2.5 M CaCl2, and 500 μL of 2x HBS was added dropwise over a monolayer of Hek293T cells seeded on a 10-cm^2^ dish. After 16 h, the medium was replaced. Thirty hours later, the medium containing virus particles was collected and passed on a 0.22-μm filter. CaLu3 cells were infected overnight and the following day selected with 1.5 μg/mL puromycin (Thermo Scientific) treatment for 10 days. Transduced cells were infected with SARS-CoV-2 on day 12 and samples collected for analysis on day 14.

### RNA extraction and reverse transcription.

Cell supernatant was collected and Maxwell RSC Viral Total Nucleic Acid purification kit was used to extract RNA from 250 μL of cell culture supernatants employing the Maxwell RSC Instrument (Promega, Madison, WI, USA). The remaining supernatant was conveniently stored at −80°C for the TCID_50_ assessment. Each well was then thoroughly washed three times with pre-warmed PBS. Cells were lysed and collected in 100 μL of RNAzol (TEL-TEST, Inc., Friendswood, TX, USA). RNA extraction was performed employing the acid guanidium-phenol-chloroform (AGPC) extraction method, as elsewhere described (88). One μg of total RNA was reversed transcribed in a final volume of 20 μL using the Reverse Transcription Kit (Promega). Target cDNA was amplified by either ddPCR or rtPCR.

### Droplet digital PCR (ddPCR) and real time PCR (rtPCR).

The QX100 Droplet Digital PCR System (Bio-Rad Laboratories) instrument was used for this study. Two μL of cDNA diluted 1:10.000 (cells) or 1:100 (cell supernatants) were mixed with commercial SARS-CoV-2 (2019-nCoV) CDC qPCR Probe Assay (IDT). Two μL of cDNA diluted 1:100 (cell extract) were mixed with commercial PrimePCR ddPCR Expression Probe Assay for CyPA/PPIA (Human, fluorophore Hex, dHsaCPE5031543, Bio-Rad). The volume of the final PCR mix was 20 μL including 10 μL of ddPCR Supermix for Probes (No dUTP) and 1 μL of the primers/fluorophore probe N1. ddPCR amplification reagents were purchased from Bio-Rad Laboratories. The droplet emulsion was thermally cycled on C1000 Touch Thermal Cycler (Bio-Rad Laboratories) instrument. Cycling conditions were 95°C for 5 min, followed by 40 cycles of amplification (94°C for 30 s and 55°C for 1 min), ending with 98°C for 10 min. The concentration of the target was calculated automatically by the QuantaSoft software version 1.7.4 (Bio-Rad Laboratories).

The LightCycler 480 instrument II (Roche, Basel, Switzerland) was used for the real-time PCR analysis. Briefly, 12.5 ng of cDNA were mixed with 10 μL of LightCycler 480 SYBR green I Master (Roche) and 150 nM final concentration of primers. rtPCR cycling conditions were: hot start at 95°C for 10 min, 45 cycles of amplification (95°C for 15 s, 60°C for 10 s, and 72°C for 20 s), final extension at 72°C for 20 s, followed by 10 min at 98°C. See Table 2 for primers used for rtPCR.

**TABLE 2:** Sequence of primers used for rtPCR in this study

Primer name	Primer sequence
GAPDH FWD	5′-cATGCCTTCTTGCCTCTTGT-3′
GAPDH REV	5′-GTTGAGGTCAATGAAGGGGTC-3′
IL1α FWD	5′-GGTTGAGTTTAAGCCAATCCA-3′
IL1α REV	5′-TGCTGACCTAGGCTTGATGA-3′
IL6 FWD	5′-GATTCAATGAGGAGACTTGCCTGG-3′
IL6 REV	5′-CTCACTACTCTCAAATCTGTTCTGG-3′
IL8 FWD	5′-CATCTCACTGTGTGTAAACATGAC-3′
IL8 REV	5′-CCTTGGCAAAACTGCACCTTCAC-3′
TNFα FWD	5′-GAGCACTGAAAGCATGATCC-3′
TNFα REV	5′-CGAGAAGATGATCTGACTGCC-3′

Results were expressed as relative expression units (nFold) to the housekeeping reference gene (GAPDH) calculated by the 2^−ΔΔCt^ method.

### Immunofluorescence.

Cells seeded, grown, and treated on glass coverslips were fixed in 4% paraformaldehyde in 0.2 M HEPES for 1 h at room temperature and permeabilized with blocking solution (PBS supplemented with 0.1% saponin, 0.5% BSA and 50 mM NH_4_Cl) for 30 min at room temperature. Cells were incubated with primary antibodies, specific Alexa 488- and 546-conjugated secondary antibodies and Hoechst 33542 diluted in blocking solution. For imaging, samples were examined using a Zeiss (Oberkochen, Germany) Imager A2 microscope, equipped with 49 DAPI (excitation 365, beam splitter FT 395, emission BP 445/50), 43 HE CY3 (excitation BP 550/25, beam splitter FT 570, emission BP 605/70), and 38 HE EGFP (excitation BP 470/40, beam splitter FT 495, emission BP 525/50) filter sets (Zeiss). Images were obtained under a 20x/0.50 Plan-Neofluar M27 objective (Zeiss), at a definition of 1388 × 1040 pixels (150 dpi), by means of a high-resolution monochromatic camera (Axiocam MRm Rev3, Zeiss), and analyzed with the Axiovision REL 4.7 software (Zeiss).

### Western blotting.

For WB analysis, cells were lysed directly in 2x Laemmli buffer in order to inactivate the virus and to be able to process them outside BSL3 area. Samples were boiled for 5 min at 95°C before loading onto the gel. Proteins were separated by SDS–PAGE and transferred onto nitrocellulose membranes (Hybond, GE Healthcare, Chalfont St. Giles, Buckinghamshire, UK). Strips containing the proteins of interest were incubated in 5% (wt/vol) BSA in TBS containing 0.1% (vol/vol) Tween 20, pH 7.4 (T-TBS), for 1 h at room temperature and then with fresh blocking buffer containing the primary antibody at its working concentration (see Table 3). After overnight incubation at 4°C, the antibodies were removed and the strips washed with T-TBS for 3 × 10 min. Strips were incubated for 1 h with the appropriate horseradish peroxidase (HRP)-conjugated secondary antibody and washed 3 × 10 min with T-TBS. WBs were developed using the chemiluminescent method (ECL, GE Healthcare) and signals acquired by ChemiDoc MP Imaging System (Bio-Rad Laboratories). Bands were quantified by densitometric analysis using the National Institutes of Health (NIH) ImageJ program. The quantification of each band was normalized using the signal of housekeeping proteins (CyPA or GAPDH) as a loading control.

**TABLE 3:** Ordering information and working conditions of antibodies used for WB in this study

Antibody	Company	Catalog	Dilution	Conditions
Spike	Genetex	632604	1:1000	Overnight,4°C
ACE2	Abcam	15348	1:1000	Overnight,4°C
CD147	Santa Cruz	53693	1:500	Overnight, 4°C
CyPA	Abcam	58144	1:500	Overnight, 4°C
GAPDH	Abcam	128915	1:40000	Overnight, 4°C
Anti-mouse HRP	Cell signaling technologies	7076	1:5000	1 hour, r.t.
Anti-rabbit HRP	Cell signaling technologies	7074	1:5000	1 hour, r.t.
