# Supplementary material for: Cyclosporine A Inhibits Viral Infection and Release as Well as Cytokine Production in Lung Cells by Three SARS-CoV-2 Variants
Source: Microbiol Spectr. 2022 Jan 5;10(1):e01504-21. doi: 10.1128/spectrum.01504-21 (PMC8729790; doi:10.1128/spectrum.01504-21)
Supplement: SUPPLEMENTAL FILE 1 — Supplemental material. Download SPECTRUM01504-21_Supp_1_seq9.pdf, PDF file, 0.4 MB [file spectrum01504-21_supp_1_seq9.pdf]

SUPPLEMENTAL INFORMATION for:

Cyclosporine A inhibits viral infection and release as well as cytokine production in lung cells by three SARS-CoV-2 variants

Claudio Fenizia[a,b], Silvia Galbiati[c], Claudia Vanetti[a,b], Riccardo Vago[d,e], Mario Clerici[a,f], Carlo Tacchetti[e,g,#] and Tiziana Daniele[g,#]

[a]Department of Pathophysiology and Transplantation, Milano University Medical School
[b]Department of Biomedical and Clinical Sciences "L Sacco", Milano University Medical School
[c]Complication of Diabetes Unit, Diabetes Research Institute, IRCCS San Raffaele Scientific Institute
[d]Urological Research Institute, IRCCS San Raffaele Scientific Institute
[e]Vita-Salute San Raffaele University
[f]IRCCS Don Carlo Gnocchi Foundation
[g]Cancer Imaging Unit, Experimental Imaging Centre, IRCCS San Raffaele Scientific Institute

SUPPLEMENTAL INFORMATION

Supplementary Fig 1: CsA reduces the number of CaLu3 cells infected by SARS-CoV-2.

SUPPL. FIG 1

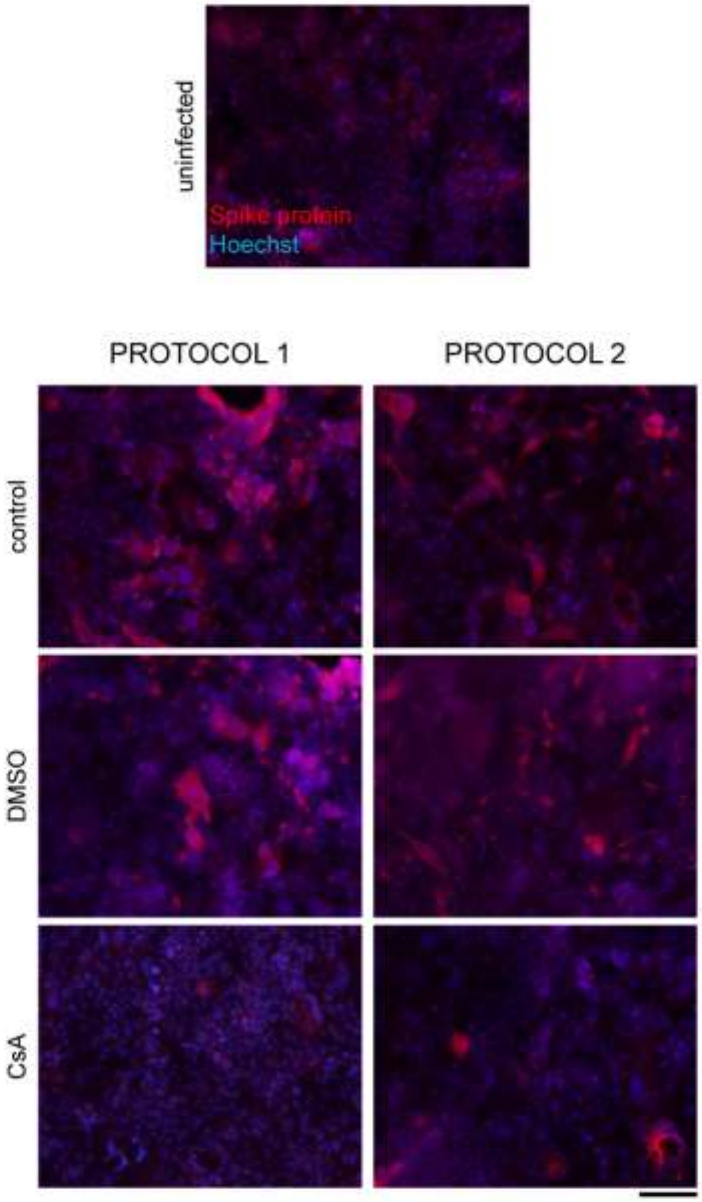

Suppl. Fig 1: CsA reduces the number of CaLu3 cells infected by SARS-CoV-2. CaLu3 cells were treated with 10 µM CsA either before (protocol 1) or after (protocol 2) SARS-CoV-2 infection. Cells were analysed 48 hpi. Cells were processed for immunofluorescence. Red: Spike protein (infected cells); blue: Hoechst (nuclei). Scale bar: 120 µm.
